# Supplementary material for: Transcriptome and Metabolome Analyses Reveal Complex Molecular Mechanisms Involved in the Salt Tolerance of Rice Induced by Exogenous Allantoin
Source: Antioxidants (Basel). 2022 Oct 18;11(10):2045. doi: 10.3390/antiox11102045 (PMC9598814; doi:10.3390/antiox11102045)
Supplement: Supplementary file 1 [file antioxidants-11-02045-s001.zip › Supplementary Tables AND figures/antioxidants-1926288-supplementary.pdf]

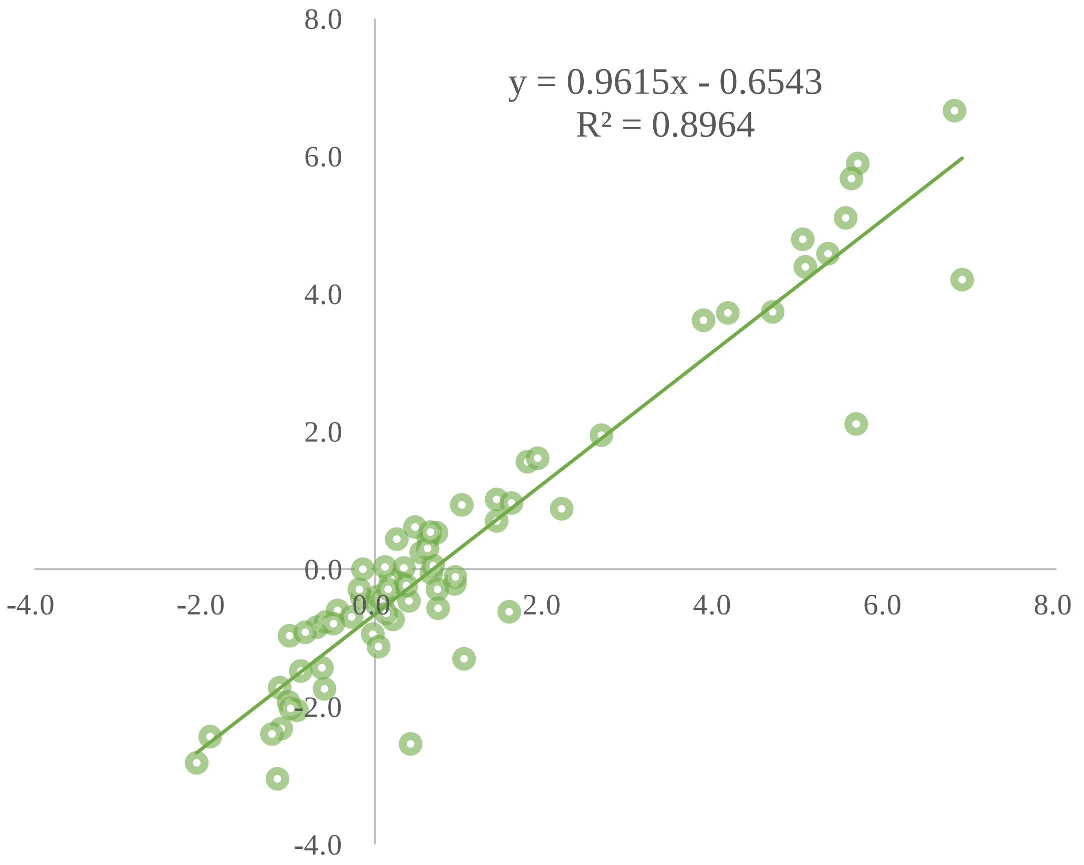


**Supplementary Figure S1.** Validation of the expression of 24 differentially expressed genes by a quantitative real-time (qRT)-PCR analysis. The genes and the corresponding qRT-PCR primers are listed in Supplementary Table 1.

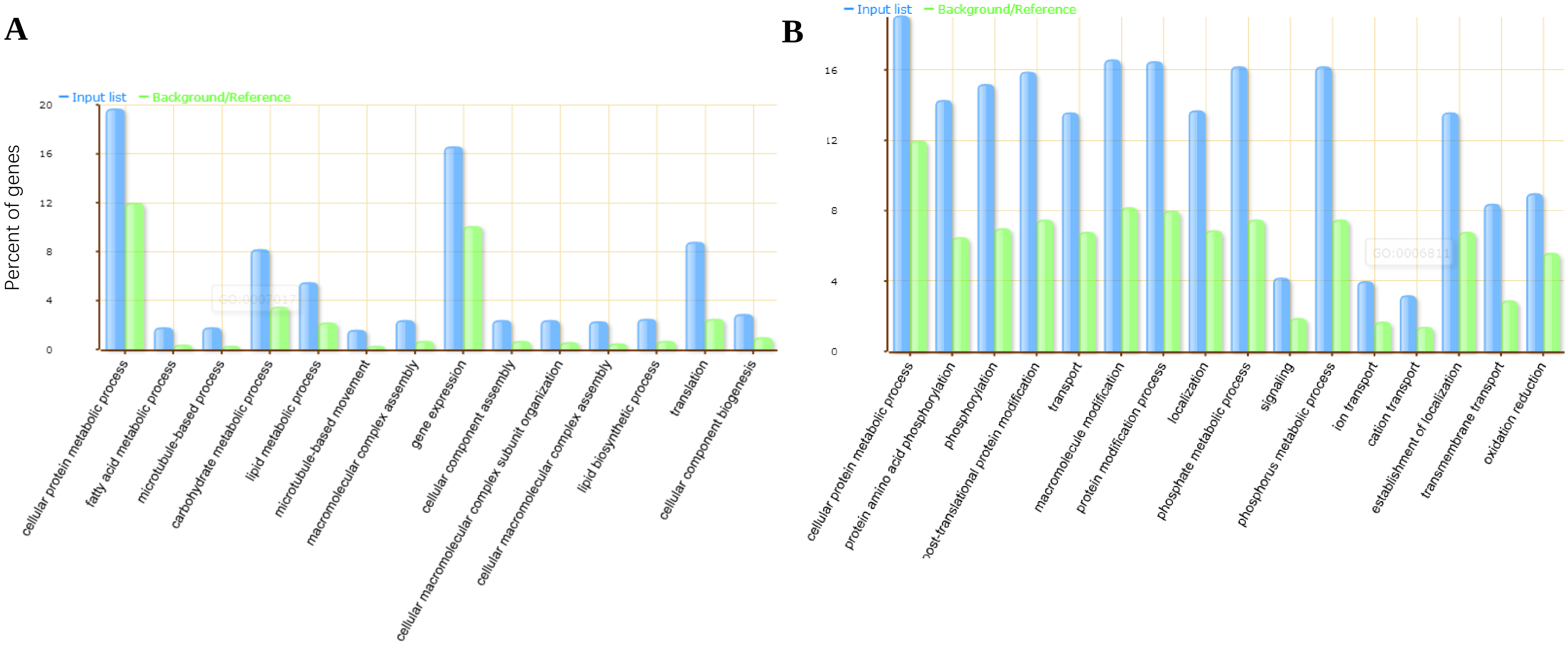


**Supplementary Figure S2.** Results of a GO enrichment analysis of the differentially expressed genes in the C18 seedlings revealed by the comparison between the allantoin treatment and the control treatment. Functional enrichment of the up-regulated genes (**A**) and down-regulated genes (**B**).

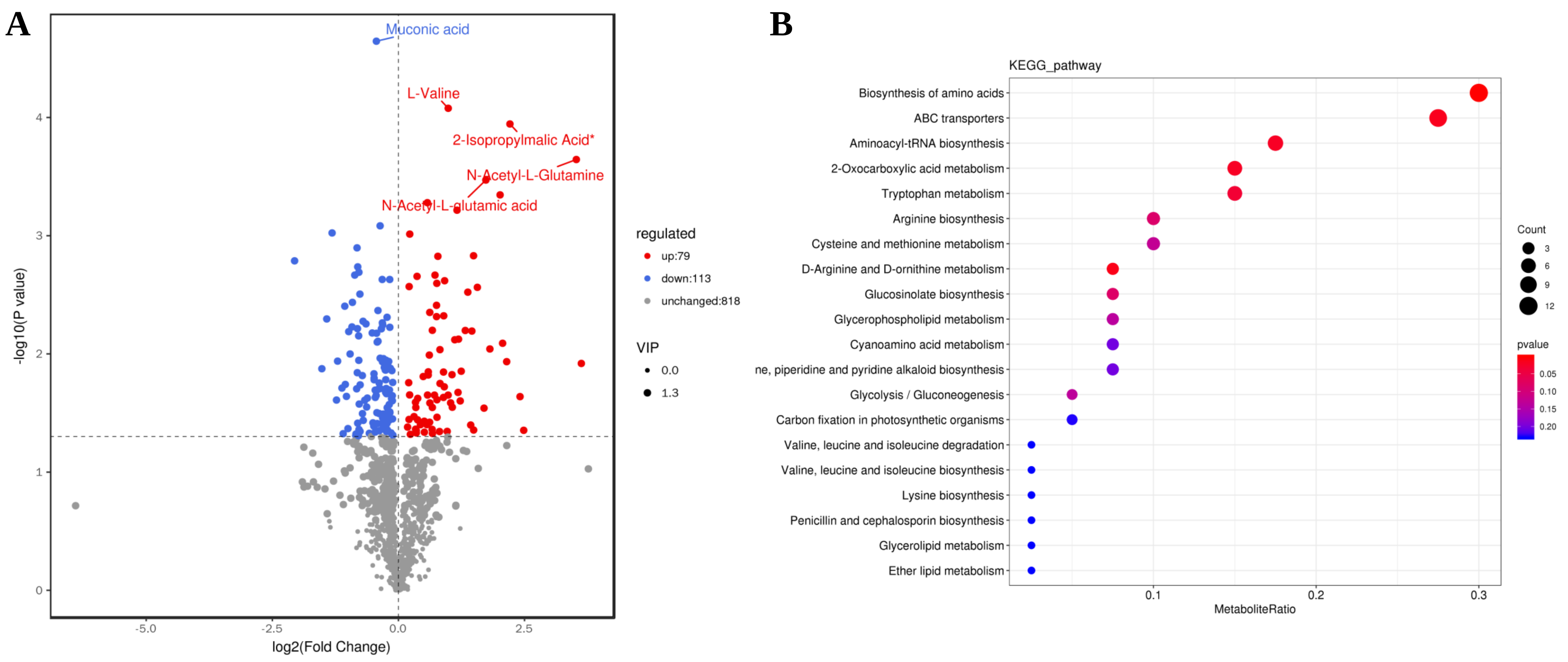


**Supplementary Figure S3**. Differentially abundant metabolites (DAMs) in the C18 seedlings revealed by the comparison between the allantoin treatment and the control treatment. Volcano diagram of the DAMs (**A**). Enriched KEGG pathways among the DAMs (**B**).
